# Managing Musculoskeletal Pain in Older Adults Through a Digital Care Solution: Secondary Analysis of a Prospective Clinical Study

**DOI:** 10.2196/49673

**Published:** 2023-08-15

**Authors:** Anabela C Areias, Dora Janela, Maria Molinos, Robert G Moulder, Virgílio Bento, Vijay Yanamadala, Steven P Cohen, Fernando Dias Correia, Fabíola Costa

**Affiliations:** 1 Sword Health, Inc Draper, UT United States; 2 Institute for Cognitive Science University of Colorado Boulder Boulder, CO United States; 3 Department of Surgery Frank H Netter School of Medicine Quinnipiac University Hamden, CT United States; 4 Department of Neurosurgery Hartford Healthcare Medical Group Westport, CT United States; 5 Departments of Anesthesiology & Critical Care Medicine, Physical Medicine and Rehabilitation, Neurology, and Psychiatry and Behavioral Sciences Johns Hopkins School of Medicine Baltimore, MD United States; 6 Departments of Anesthesiology and Physical Medicine and Rehabilitation Uniformed Services University of the Health Sciences Bethesda, MD United States; 7 Neurology Department Centro Hospitalar e Universitário do Porto Porto Portugal

**Keywords:** aged, digital therapy, eHealth, musculoskeletal conditions, older adults, pain, physical therapy, telehealth, telerehabilitation

## Abstract

**Background:**

Aging is closely associated with an increased prevalence of musculoskeletal conditions. Digital musculoskeletal care interventions emerged to deliver timely and proper rehabilitation; however, older adults frequently face specific barriers and concerns with digital care programs (DCPs).

**Objective:**

This study aims to investigate whether known barriers and concerns of older adults impacted their participation in or engagement with a DCP or the observed clinical outcomes in comparison with younger individuals.

**Methods:**

We conducted a secondary analysis of a single-arm investigation assessing the recovery of patients with musculoskeletal conditions following a DCP for up to 12 weeks. Patients were categorized according to age: ≤44 years old (young adults), 45-64 years old (middle-aged adults), and ≥65 years old (older adults). DCP access and engagement were evaluated by assessing starting proportions, completion rates, ability to perform exercises autonomously, assistance requests, communication with their physical therapist, and program satisfaction. Clinical outcomes included change between baseline and program end for pain (including response rate to a minimal clinically important difference of 30%), analgesic usage, mental health, work productivity, and non–work-related activity impairment.

**Results:**

Of 16,229 patients, 12,082 started the program: 38.3% (n=4629) were young adults, 55.7% (n=6726) were middle-aged adults, and 6% (n=727) were older adults. Older patients were more likely to start the intervention and to complete the program compared to young adults (odds ratio [OR] 1.72, 95% CI 1.45-2.06; *P*<.001 and OR 2.40, 95% CI 1.97-2.92; *P*<.001, respectively) and middle-aged adults (OR 1.22, 95% CI 1.03-1.45; *P*=.03 and OR 1.38, 95% CI 1.14-1.68; *P*=.001, respectively). Whereas older patients requested more technical assistance and exhibited a slower learning curve in exercise performance, their engagement was higher, as reflected by higher adherence to both exercise and education pieces. Older patients interacted more with the physical therapist (mean 12.6, SD 18.4 vs mean 10.7, SD 14.7 of young adults) and showed higher satisfaction scores (mean 8.7, SD 1.9). Significant improvements were observed in all clinical outcomes and were similar between groups, including pain response rates (young adults: 949/1516, 62.6%; middle-aged adults: 1848/2834, 65.2%; and older adults: 241/387, 62.3%; *P*=.17).

**Conclusions:**

Older adults showed high adherence, engagement, and satisfaction with the DCP, which were greater than in their younger counterparts, together with significant clinical improvements in all studied outcomes. This suggests DCPs can successfully address and overcome some of the barriers surrounding the participation and adequacy of digital models in the older adult population.

## Introduction

The US population over 65 years of age is forecast to double in the coming decades, from 49.2 million in 2016 to 94.7 million people in 2060, depicting aging as a major driver of changes in US health care systems [1]. Aging is associated with an increased likelihood of developing musculoskeletal conditions [2-5], with around 40% to 60% of older adults reporting persistent musculoskeletal pain [6]. Older adults contribute to 35.2% of the US $381 billion annual spending in this domain [7]. Musculoskeletal disorders elevate the risk of developing comorbidities [8] and increase the odds of mortality [9] in older adults as a result of decreased physical activity, which increases falls and frailty, poor mental health, sleep disturbances, and overall impaired quality of life [2,3,10-13].

Current guidelines advocate for exercise-based physical therapy as the mainstay intervention in musculoskeletal care [14-16]. Telerehabilitation emerged to address barriers associated with conventional physical therapy, thereby improving access to care by mitigating provider shortages, travel and time constraints, and obviating concerns about infection during the COVID-19 pandemic [17]. Despite a general acceptance of telerehabilitation, older adults face specific barriers and concerns associated with digital programs [18,19]. These are related to accessing and being comfortable technology, internet accessibility, perception of a lack of personal connection in digital care, and perceived insufficient effectiveness of remote interventions. Thus, it is particularly important to frame the development of interventions acknowledging generational needs. Helping older adults become more tech-savvy has been shown to improve their health and overall quality of life, as it improves access to information and to community while promoting self-efficacy in daily life [20]. Moreover, the internet usage gap between those who are older than 65 years and younger individuals has narrowed in the past decade [1], providing an opportunity to leverage digital health as a scalable solution that will benefit older adults. Herein, we describe a patient-centered multimodal digital care program (DCP) combining exercise with education and cognitive behavioral therapy (CBT) that has been validated for several acute and chronic musculoskeletal conditions [21-25]. This program was designed to maximize adherence, acknowledging each participant’s unique needs. This study aimed to investigate whether the known barriers and concerns of older patients impacted their participation in or engagement with a DCP, or the observed clinical outcomes, in comparison with younger individuals. This secondary analysis hypothesizes that regardless of age, all patients will experience comparable levels of engagement and significant improvements in all clinical outcomes.

## Methods

### Study Design

This is a secondary analysis of a single-arm investigation into clinical and engagement-related outcomes of patients with musculoskeletal conditions following a DCP delivered between June 18, 2020, and August 3, 2022.

### Study Population

Inclusion criteria were US adult (≥18 years of age) beneficiaries of employer health plans with the presence of musculoskeletal pain either in the ankle, elbow, hip, knee, low back, neck, shoulder, wrist, or hand, and duration of pain of >12 weeks. Eligible individuals were invited to apply to Sword Health’s DCP (Draper, Utah) through a dedicated enrollment website. Exclusion criteria include health conditions incompatible with at least 20 minutes of light to moderate exercise, ongoing cancer treatment, and the presence of signs or symptoms indicative of serious pathology (eg, rapid progressive motor weakness or sensory alterations, or bowel or bladder dysfunction). All participants provided informed consent. Participants who skipped exercise sessions for 28 consecutive days were considered dropouts.

### Intervention

The intervention consisted of exercise, education, and CBT administered for up to 12 weeks, depending on each patient’s condition. During onboarding, patients selected a certified doctor of physical therapy (DPT) according to their preferences, who was responsible for tailoring and monitoring the program according to the patient’s goals. Each patient received a Food and Drug Administration–listed class II medical device that included a tablet with a mobile app (already installed and ready to use), which displayed exercises and provided real-time video and audio biofeedback on exercise execution through either the use of motion trackers or the tablet’s camera. It was recommended that patients perform 3 sessions per week. Exercise data were stored in a cloud-based portal that enabled asynchronous and remote monitoring by the DPT. Condition-specific education and CBT were made available through written articles, audio content, and interactive modules focused on health literacy, pain self-management skills, and mental health [14-16].

The DCP was designed to minimize barriers for those less comfortable with technology and to build trust and commitment from the start. This included an on-call onboarding assistant who was available to help fill out the onboarding form and answer any questions regarding the program’s journey. Onboarding assistance was also provided through the enrollment web chat room. Tablet app design followed best practices for acknowledging older adults’ use [26] (eg, white spaces between content, allowing to adjust font size and audio volume). The time between exercises could be adjusted to age-appropriate rhythms. Continuous technical support was available to troubleshoot any issues across the intervention (either related to tablet, sensors, or connectivity). A set-up booklet was provided to guide tablet initiation and Wi-Fi connection. A Wi-Fi hotspot was provided to those lacking an internet connection. A personal connection with the DPT was fostered through the onboarding video call and a built-in secure chat on a smartphone app. This allowed for rapport development between DPTs (frequent outreach to provide motivation and feedback on evolution) and patients (who could share ongoing questions and concerns).

### Outcomes

Assessment surveys collected at baseline, 4, 8, and 12 weeks were used to analyze mean changes in clinical outcomes between baseline and program end. Engagement data were collected from the cloud-based portal. Table 1 describes the studied outcomes.

**Table 1:** Description of the assessed outcomes.

Outcome measure		Outcome description
**Engagement**		
	Assistance requests	Amount of support requests during enrollment, onboarding, app installment, member account set-up, and participation
	Exercise performance	Corresponds to the sum of correct movements divided by the sum of total movements (independently if correct or incorrect) for each session
	Sessions per week	Mean number of sessions performed per week
	Total time on sessions	Total time spent exercising during the intervention
	Total articles read	Number of articles read during the intervention
	Total messages sent by the member	Number of text messages sent by the patient to the DPT^a^
	Satisfaction	Evaluated through the question: “On a scale from 0 to 10, how likely is it that you would recommend this intervention to a friend or neighbor?”
**Clinical**		
	Numerical Pain Rating Scale	“Please rate your average pain over the past 7 days from 0 (no pain at all) to 10 (worst pain imaginable).” A 30% or greater decrease was considered to represent a “Minimal clinically important difference (MCID)” [27]
	Mental health	Anxiety was assessed by the GAD-7^b^ (range 0-21) [28], and depression was assessed by the PHQ-9^c^ (range 0-27) [29], in which higher scores denote worse outcomes
	WPAI^d^	Collected within employed population to assess overall work impairment (WPAI overall), presenteeism (WPAI work), absenteeism (WPAI time), and activities impairment (WPAI activity) [30], with higher scores denoting poorer outcomes
	Analgesics intake	Consumption of analgesics (either over-the-counter or prescribed) for the treated condition (binary response)

^a^DPT: doctor of physical therapy.

^b^GAD-7: Generalized Anxiety Disorder 7-item scale.

^c^PHQ-9: Patient Health Questionnaire 9-item scale.

^d^WPAI: Work Productivity and Activity Impairment Questionnaire.

### Statistical Analysis

Participants were categorized into 3 age groups: ≤44 years old (young adults), 45-64 years old (middle-aged adults), and ≥65 years old (older adults). The threshold used to identify older adults is in accordance with age classifications established by the World Health Organization [31] and the US Census, while the threshold to differentiate young and middle-aged adults was based on previous reports from the Centers for Disease Control and Prevention [32,33]. Demographics and clinical outcomes at baseline and engagement metrics were compared between groups using a 1-way ANOVA with Bonferroni correction or chi-square test. Distance to health care facilities was calculated using each patient’s geo-coordinates cross-referenced with the geographic location of health care resources (filtered for clinics, doctors, hospitals, and rehabilitation units) [34,35].

A multiple-group latent growth curve analysis (mLGCA) following an intention-to-treat approach was used to assess clinical outcome changes at the program end as well as exercise performance across the program. LGCA is a structural equation model [36] that provides estimates of overall change based on individual trajectories using time as a continuous variable. Key advantages of LGCA include providing a measure of fitness and addressing missing data through full information maximum likelihood [37]. mLGCA allows the creation of separate models for different groups, accounting for unbalanced group size while simultaneously permitting intergroup comparisons. An analysis focused on patients with minimally significant baseline impairment in the various domains was performed: ≥5 points for Generalized Anxiety Disorder 7-item scale (GAD-7) and Patient Health Questionnaire 9-item scale (PHQ-9) [28,29], and >0 for Work Productivity and Activity Impairment Questionnaire (WPAI; overall, work, time, and activity). A robust sandwich estimator was used for standard errors. Gender, BMI, race or ethnicity (White, non-White, and prefer not to specify), rurality (rural vs urban [38]), and symptomatic anatomical areas (upper limb, lower limb, and spine) were used as covariates for all the above-mentioned models.

An adjusted ordinal regression analysis was performed to longitudinally assess the latent distribution of analgesic consumption until the program ended within and between age groups. An adjusted odds ratio (OR) for being a program starter, being a completer, and reaching the minimum clinically important difference for pain was calculated using binary logistic regression.

Since education levels were considered a robust and consistent predictor of eHealth literacy [39], the impact of education levels (lower education: less than high school diploma, high school diploma, and some college vs higher education: bachelor’s or graduate degree) on engagement outcomes was evaluated among older adults through mLGCA. All statistical analyses were conducted using commercially available software (SPSS v22; IBM Corp) and R (version 4.2.2, R Foundation for Statistical Computing). The level of significance was set at *P*<.05 for all 2-sided hypothesis tests.

### Ethics Approval

The trial was prospectively approved (New England IRB number 120190313) and registered on ClinicalTrials.gov (NCT04092946) on September 17, 2019.

## Results

### Overview

**Figure 1:**
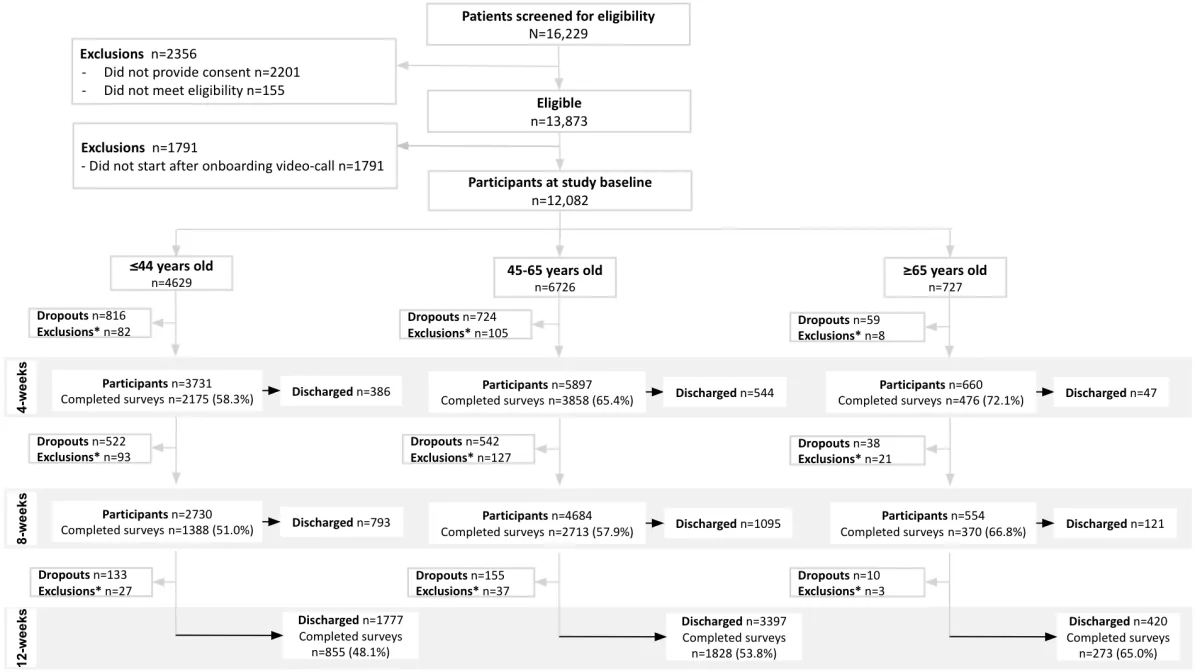
Flowchart of the study stratified by age following the CONSORT (Consolidated Standards of Reporting Trials) guidelines. *Exclusions unrelated to the clinical condition.

From a total of 16,229 patients, 12,082 (74.4%) started the study, of which 4629 (38.3%) were young adults (≤44 years old), 6726 (55.7%) were middle-aged adults (45-64 years old), and 727 (6%) were older adults (≥65 years old; Figure 1 and Table 2).

The likelihood to start the intervention (ie, engaging with exercise sessions) was higher among older adults compared to young adults (OR 1.72, 95% CI 1.45-2.06; *P*<.001), and middle-aged adults (OR 1.22, 95% CI 1.03-1.45; *P*=.03). The proportion of those requesting assistance (in the scope of the enrollment, onboarding, app install, member account registration, and set-up questions) was higher for older adults (138/727, 19%) versus middle-aged adults (1031/6726, 15.3%) and young adults (481/4629, 10.4%; *P*<.001), with similar assistance requests per person between groups (mean 1.2, SD 0.5 requests per person in middle-aged adults vs mean 1.1, SD 0.4 requests per person in young adults and mean 1.1, SD 0.4 requests per person for older adults; *P*<.001). The older adults group was also more likely to complete the program than the young (OR 2.40, 95% CI 1.97-2.92; *P*<.001) and middle-aged adults (OR 1.38, 95% CI 1.14-1.68; *P*=.001) groups.

**Table 2:** Cohort demographic characteristics stratified by age groups. Missing values: BMI (n=23); geographic location (n=4); and distance to health facilities within 6 miles (n=25).

Characteristics		Age groups			*P* value
		Young adults (≤44 years old; n=4629)	Middle-aged adults (45-64 years old; n=6726)	Older adults (≥65 years old; n=727)	
Age (years), mean (SD)		35.9 (5.7)	54.5 (5.6)	67.3 (3.1)	<.001
**Gender, n (%)**					<.001
	Woman	2587 (55.9)	4005 (59.5)	352 (48.4)	
	Man	2011 (43.4)	2708 (40.3)	372 (51.2)	
	Nonbinary	25 (0.5)	10 (0.1)	2 (0.3)	
	Other	0 (0)	1 (0)	1 (0.1)	
	Prefers not to answer	6 (0.1)	2 (0)	0 (0)	
BMI (kg/m^2^), mean (SD)		28.4 (6.7)	29.7 (6.7)	29 (5.8)	<.001
**BMI category (kg/m^2^), n (%)**					<.001
	Underweight (<18.5)	51 (1.1)	39 (0.6)	6 (0.8)	
	Normal (18.5-25)	1576 (34)	1663 (24.7)	165 (22.7)	
	Overweight (25-30)	1524 (32.9)	2266 (33.7)	296 (40.7)	
	Obese (30-40)	1169 (25.3)	2199 (32.7)	224 (30.8)	
	Morbidly obese (>40)	305 (6.6)	542 (8.1)	34 (4.7)	
**Race and ethnicity, n (%)**					<.001
	Asian	460 (9.9)	458 (6.8)	32 (4.4)	
	Black	343 (7.4)	559 (8.3)	35 (4.8)	
	Hispanic	444 (9.6)	462 (6.9)	29 (4)	
	Non-Hispanic White	2078 (44.9)	3384 (50.3)	391 (53.8)	
	Other	139 (3)	108 (1.6)	1 (0.1)	
	Not available or prefers not to specify	1165 (25.2)	1755 (26.1)	239 (32.9)	
**Employment status, n (%)**					<.001
	Employed	4278 (92.4)	6139 (91.3)	577 (79.4)	
	Not employed	233 (5)	426 (6.3)	138 (19)	
	Not available or prefers not to answer	118 (2.5)	161 (2.4)	12 (1.7)	
**Education level, n (%)**					<.001
	Less than high school diploma	25 (0.5)	46 (0.7)	5 (0.7)	
	High school diploma	266 (5.7)	501 (7.4)	68 (9.4)	
	Some college	835 (18)	1518 (22.6)	155 (21.3)	
	Bachelor’s degree	1694 (36.6)	2184 (32.5)	208 (28.6)	
	Graduate degree	1040 (22.5)	1353 (20.1)	183 (25.2)	
	Prefers not to answer or is not available	769 (16.6)	1124 (16.7)	108 (14.9)	
**Geographic location, n (%)**					<.001
	Urban	4182 (90.4)	5918 (88)	631 (86.8)	
	Rural	446 (9.6)	805 (12)	96 (13.2)	
**Minimum distance to nearest health care facilities in miles**					<.001
	Median (IQR)	2.1 (3.5)	2.5 (4.2)	2.6 (4.1)	
	Mean (SD)	4.2 (6.1)	4.9 (6.6)	5.1 (7)	
**Number of health care facilities located within 6-mile radius of residence**					<.001
	Median (IQR)	5.00 (14)	4.00 (8)	3.00 (8)	
**Symptomatic anatomical area, n (%)**					<.001
	Ankle	229 (4.9)	263 (3.9)	20 (2.8)	
	Elbow	91 (2)	175 (2.6)	7 (1)	
	Hip	399 (8.6)	683 (10.2)	97 (13.3)	
	Knee	599 (12.9)	1097 (16.3)	153 (21)	
	Low back	1937 (41.8)	2359 (35.1)	269 (37)	
	Neck	538 (11.6)	661 (9.8)	57 (7.8)	
	Shoulder	666 (14.4)	1229 (18.3)	109 (15)	
	Wrist or hand	170 (3.7)	259 (3.9)	15 (2.1)	

### Baseline Characteristics

The older adults group was more balanced gender-wise compared to other age groups, which contained a greater proportion of women (Table 2). Young and middle-aged cohorts had lower BMI levels and included significantly more people of color than older adults (Table 2). Although the majority of older adults were employed (79.4%), the group also had the highest percentage of nonemployed participants (19% vs 6.3% and 5%; *P*<.001), which was primarily due to the high percentage of retirees (106/138). Young adults reported significantly higher education levels than middle-aged and older adults (Table 2). Older adults mainly resided in urban areas but also had the highest percentage (13.2%) of patients situated in rural areas compared to young (9.6%) and middle-aged adults (12%; *P*<.001). Older adults lived farther away from health care facilities, with fewer providers within a 6-mile radius compared to other groups (Table 2).

The most reported symptomatic anatomical areas across groups were the low back, knee, and shoulder (Table 2). Pain scores were significantly higher in older (mean 4.83, SD 2.0) and middle-aged adults (mean 4.90, SD 2.0) compared to young adults (mean 4.48, SD 1.9; *P*<.001; Table 3). A commensurate trend was observed for analgesic consumption (34.3% of older adults vs 27.1% in middle-aged adults vs 16% in young adults; *P*<.001). Among those who reported at least mild anxiety or depression symptoms at baseline, older adults had lower levels of anxiety (mean 7.98, SD 3.6 vs mean 8.54, SD 3.9 in middle-aged adults and mean 9.2, SD 4.1 in young adults; *P*<.001), and depression (mean 8.12, SD 3.4 vs mean 9.07, SD 4.2 in middle-aged and mean 9.66, SD 4.5 in young adults; *P*<.001; Table 3). A significantly higher proportion of young adults (2429/4278, 56.8%) reported overall productivity impairment at baseline versus middle-aged (3217/6139, 52.4%) and older adults (281/577, 48.7%; *P*<.001; Table 3). However, similar average work productivity and activity impairment scores were observed between groups (Table 3). Presenteeism was particularly an issue for young adults (WPAI work: mean 29, SD 19; *P*=.02), while absenteeism was mainly reported by older adults still in the workforce compared to other age categories (WPAI time: mean 40, SD 40.6; *P*=.002; Table 3).

**Table 3:** Clinical characteristics at baseline stratified by age. For unfiltered cases, see Table S1 in Multimedia Appendix 1.

Outcomes	Age group						*P* value
	Young adults (≤44 years old)		Middle-aged adults (45-64 years old)		Older adults (≥65 years old)		
	Patients, n	Mean (SD)	Patients, n	Mean (SD)	Patients, n	Mean (SD)	
Pain	4629	4.48 (1.9)	6726	4.90 (2.0)	727	4.83 (2.0)	<.001
GAD-7^a^ score of ≥5	1778	9.15 (4.1)	1815	8.54 (3.9)	133	7.98 (3.6)	<.001
PHQ-9^b^ score of ≥5	1292	9.66 (4.5)	1410	9.07 (4.2)	136	8.12 (3.4)	<.001
WPAI^c^-Overall score of >0	2429	31.4 (21.8)	3217	30.9 (22.0)	281	29.7 (23.5)	.42
WPAI-Work score of >0	2363	29 (19.0)	3090	28.4 (18.8)	260	25.7 (17.6)	.02
WPAI-Time score of >0	477	23.8 (28.0)	595	26 (30.5)	46	40 (40.6)	.002
WPAI-Activity score of >0	3532	35 (21.5)	5109	35.7 (22.5)	536	35.2 (22.3)	.31
Analgesic intake (binary), n (%)	741 (16)	N/A^d^	1821 (27.1)	N/A	249 (34.3)	N/A	<.001

^a^GAD-7: Generalized Anxiety Disorder 7-item scale.

^b^PHQ-9: Patient Health Questionnaire 9-item scale.

^c^WPAI: Work Productivity and Activity Impairment Questionnaire.

^d^N/A: not applicable.

### Engagement Outcomes

#### Overview

Older adults completed significantly more sessions than the other groups (sessions per week: mean 3.1, SD 1.2 for older adults vs mean 2.4, SD 0.9 for young adults, and mean 2.7, SD 1.1 for middle-aged adults; *P*<.001). Older adults also dedicated more overall time to sessions (mean 698.5, SD 740.4 minutes) than young (mean 320.6, SD 354.7 minutes; *P*<.001) and middle-aged adults (mean 473.9, SD 524.6 minutes; *P*<.001).

**Figure 2:**
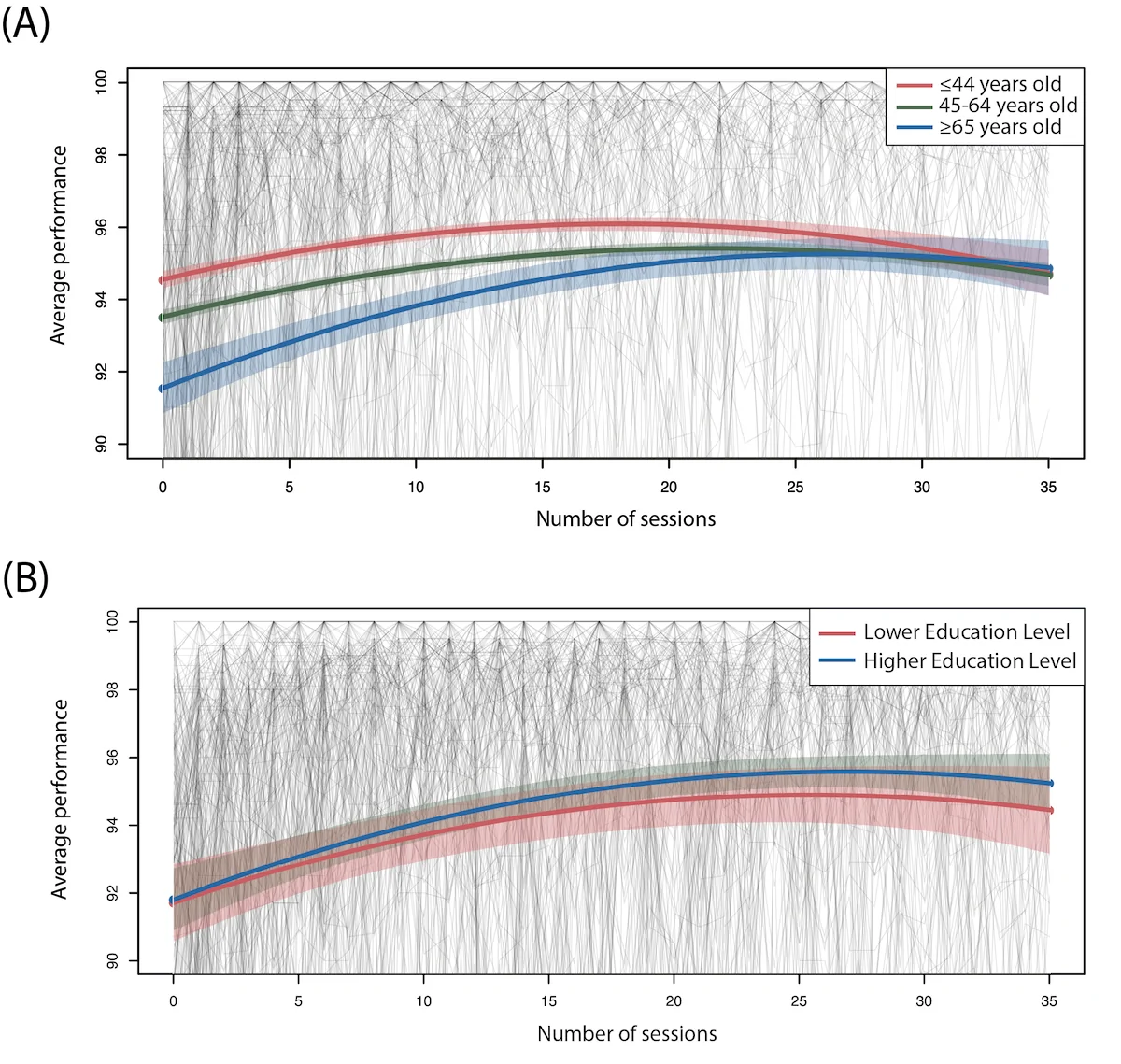
Engagement outcomes. (A) Average exercise performance trajectories broken down by age group. (B) Average performance for older adults stratified by education attainment. Shadowing indicates each trajectory’s confidence interval, while individual trajectories are depicted with lighter gray lines.

Regarding the learning curve for correctly performing the proposed exercises, all groups attained high exercise performance (>90%) at the intervention start, with older adults performing at significantly lower levels than the other cohorts (intercept: 91.5, 95% CI 90.8-92.2 vs 93.5, 95% CI 93.3-93.7 for middle-aged adults and 94.5, 95% CI 94.3-94.8 for young adults; *P*<.001 for all combinations; Figure 2A and Table S2 in Multimedia Appendix 1 [40,41]). However, the difference between older and middle-aged exercise performance disappeared by session 20 (Figure 2A and Tables S2 and S3 in Multimedia Appendix 1). The leveling effect observed toward the intervention’s end was not statistically significant between groups. Older adults read on average more pieces of education than other groups (mean 3.9, SD 6.7 vs mean 2.2, SD 4.3 young adults; *P*<.001 vs mean 3.3, SD 6.0 middle-aged adults; *P*=.005).

Both older adults (mean 12.6, SD 18.4) and middle-aged adults (mean 11.8, SD 16.7) sent significantly more text messages with the DPT than young adults (mean 10.7, SD 14.7; *P*=.02 and *P*=.004, respectively). Total satisfaction with the program was high, with older adults (mean 8.7, SD 1.9) and middle-aged adults (mean 8.8, SD 1.7) being significantly more satisfied with the program than younger patients (mean 8.5, SD 1.8; *P*<.001).

#### Subgroup Analysis: Impact of Education Level on Older Adults’ Engagement

Among older adults, those with lower education levels spent a similar amount of time on sessions (mean 640.5, SD 599.3 vs mean 649.3, SD 705.5; *P*=.09) and participated in a similar number of sessions per week (mean 3.0, SD 1.2 vs mean 3.1, SD 1.2; *P*=.10) as those with higher education levels. Similar numbers of educational resources were viewed (mean 3.5, SD 6.1 vs mean 3.7, SD 6.6; *P*=.38) and messages were sent to the DPT (mean 13.5, SD 20.1 vs mean 11.8, SD 16.5; *P*=.32) in the older cohort regardless of education level. Exercise performance trajectories were not influenced by education level (Figure 2B and Table S4 in Multimedia Appendix 1). Overall satisfaction was similar between groups (mean 8.7, SD 1.8 for the lower education subgroup vs mean 8.9, SD 1.7 for the high education subgroup; *P*=.25). 

### Clinical Outcomes

Clinical outcomes are presented in Table 4. The mLGCA model’s estimates and fitness are presented in Tables S5 and S6 in Multimedia Appendix 1, respectively, showing a good fit.

**Table 4:** Program end and estimated outcome mean change for each age category.

Outcome measure		Young adults (≤44 years old; n=4629), mean (95% CI)	Middle-aged adults (45-64 years old; n=6726), mean (95% CI)	Older adults (≥65 years old; n=727), mean (95% CI)
**Pain**				
	Program end	1.90 (1.62 to 2.18)	2.09 (1.91 to 2.26)	2.53 (1.97 to 3.08)
	Mean change	2.37 (2.08 to 2.66)	2.62 (2.43 to 2.80)	2.11 (1.53 to 2.69)
**GAD-7^a^ score of ≥5**				
	Program end	3.82 (2.73 to 4.90)	3.99 (3.03 to 4.95)	4.90 (3.06 to 6.74)
	Mean change	5.21 (4.15 to 6.28)	4.26 (3.32 to 5.22)	3.10 (0.98 to 5.22)
**PHQ-9^b^ score of ≥5**				
	Program end	4.25 (2.74 to 5.76)	3.13 (2.16 to 4.10)	4.79 (2.47 to 7.11)
	Mean change	4.97 (3.53 to 6.42)	4.94 (3.93 to 5.94)	2.10 (–0.70 to 4.90)
**WPAI^c^**-**Overall score of >0**				
	Program end	13.71 (9.52 to 17.90)	10.73 (7.83 to 13.62)	17.98 (7.15 to 28.81)
	Mean change	16.01 (11.87 to 20.15)	18.29 (15.26 to 21.32)	7.12 (0 to 17.65)
**WPAI-Work score of >0**				
	Program end	12.12 (8.33 to 15.90)	8.59 (6.22 to 10.96)	12.58 (5.89 to 19.26)
	Mean change	14.70 (10.85 to 18.54)	17.82 (15.30 to 20.34)	9.77 (3.21 to 16.33)
**WPAI-Time score of >0^d^**				
	Program end	9.50 (5.7 to 13.28)	8.12 (5.24 to 11.00)	13.45 (2.93 to 24.00)
	Mean change	14.13 (10.06 to 18.20)	17.84 (14.45 to 21.21)	23.88 (13.64 to 34.12)
**WPAI-Activity score of >0**				
	Program end	11.25 (8.46 to 14.04)	12.27 (10.17 to 14.37)	12.20 (6.46 to 17.94)
	Mean change	20.69 (17.66 to 23.73)	19.34 (17.11 to 21.57)	13.62 (7.03 to 20.20)

^a^GAD-7: Generalized Anxiety Disorder 7-item scale.

^b^PHQ-9: Patient Health Questionnaire 9-item scale.

^c^WPAI: Work Productivity and Activity Impairment Questionnaire.

^d^WPAI Time results were yielded from an unconditional model due to poor model fitness when adjusting for the covariates.

#### Pain

All age groups experienced significant reductions in pain by program end (Table 4), with no statistically significant differences between them (young adults: 2.37, 95% CI 2.08-2.66; middle-aged adults: 2.62, 95% CI 2.43-2.80; and older adults: 2.11, 95% CI 1.53-2.69; *P* values in Table S7 in Multimedia Appendix 1). Response rate did not differ across groups (young adults: 949/1516, 62.6%; middle-aged adults: 1848/2834, 65.2%; and older adults: 241/387, 62.3%; *P*=.17), when considering a 30% minimal clinically important difference for pain [27].

#### Analgesic Consumption

All groups reduced analgesic consumption by the program’s end. Using intention-to-treat analysis, a lower probability of analgesic intake at the program’s end was similar across groups (mean change in young adults group: –0.040; *P*<.001; middle-aged adults group: –0.056; *P*<.001; and older adults group: –0.091; *P*<.001; Table S8 and Figure S1 in Multimedia Appendix 1).

#### Mental Health

Despite different mental distress levels (GAD-7 and PHQ-9 scores of ≥5) at baseline (Table 3), all groups showed significant and similar improvements at the intervention’s end (*P*<.001; Table 4, Table S7 in Multimedia Appendix 1). The observed end scores indicated the absence of relevant anxiety (young adults: 3.82, 95% CI 2.73-4.90; middle-aged adults: 3.99, 95% CI 3.03-4.95; and older adults: 4.90, 95% CI 3.06-6.74) [28], and depression symptoms at program end (young adults: OR 4.25, 95% CI 2.74-5.76; adults: OR 3.13, 95% CI 2.16-4.10; and older adults: OR 4.76, 95% CI 2.47-7.11) [29].

#### Productivity

Recovery in overall productivity was significant and similar between groups (mean changes for young adults 16.01, 95% CI 11.87-20.15; middle-aged adults 18.29, 95% CI 15.26-21.32; and older adults 7.12, 95% CI 0-17.65; Table 4 and *P* values in Table S7 in Multimedia Appendix 1). Older adults reported similar presenteeism recovery to young adults (9.77, 95% CI 3.21-16.33 vs 14.70, 95% CI 10.85-18.54, respectively; *P*=.20), but slightly lower than middle-aged adults (vs 17.81, 95% CI 15.30-20.34; *P*=.02; Table 4 and Table S7 in Multimedia Appendix 1). The older adults group reported a high improvement in absenteeism (23.88, 95% CI 13.64-34.12), which was not significantly different from the other groups (14.13, 95% CI 10.06-18.20, *P*=.08 in young adults and 17.84, 95% CI 14.45-21.21, *P*=.27 in middle-aged adults; Table S7 in Multimedia Appendix 1). All groups recovered from the impairment in non–work-related activities to the same extent (Table S7 in Multimedia Appendix 1).

## Discussion

### Main Findings

Older adults may face age-specific barriers and concerns when considering digital musculoskeletal care. This study aimed to investigate whether these barriers and concerns impacted their participation in or engagement with a DCP or the observed clinical outcomes in comparison with younger individuals. Here, among those who applied to the program, older adults were more likely to start the intervention. Although they requested more technical assistance and exhibited lower initial exercise performance, the performance gap shortened over time, disappearing after 20 sessions. Overall, engagement was higher among older adults. The adherence to exercise and education and the frequent communication with the DPT suggest older adults felt comfortable with the technology and were able to establish a therapeutic relationship. Engagement outcomes were not influenced by education level, which was used as a proxy for digital literacy. Significant and similar clinical improvements in pain (with similar response rate), mental health, analgesic consumption, and productivity were observed across age groups, reinforcing the relevance of the program regardless of age. Overall, this study supports the delivery of digital musculoskeletal care to older adults.

### Comparison With Previous Research

Older adults account for 16% of the US population [42], whose distribution in terms of race and ethnicity [42], rurality [43], and employment [42] matches the older adult cohort herein described. 

#### Comfort With Technology

Health equity considerations highlight the importance of developing interventions that specifically address the barriers and concerns felt by older patients. Evidence suggests that musculoskeletal digital programs are feasible in this population [44-47]. In this study, we observed higher adoption than previously reported for older adults [45,46], as well as a higher likelihood of starting the intervention than their younger counterparts, suggesting that the possible distrust phenomenon was overcome in this particular cohort.

Although a higher number of older adults asked for technical assistance, the mean requests per patient were similar across groups. At the intervention start, older adults had lower exercise performance than younger groups, despite starting at a high score. Importantly, older patients were able to learn and improve their performance, challenging the myth that older adults are less capable of using technology. This is further reinforced by the similar engagement metrics observed regardless of education levels, although the older adult cohort reported a slightly higher proportion of those with higher education (bachelor’s degree or higher) than the US population [42].

The tailored exercise program with continuous feedback and monitoring may have empowered patients to exercise [48,49], positively impacting their self-efficacy and motivation to adhere to the intervention, as previously suggested [50]. Older adults were more adherent than other age groups, as shown by the higher number of executed sessions, time dedicated to sessions, and completion rates, in accordance with previous literature [44]. However, older adults were on average located farther away from health care facilities, which bolsters the rationale for using a DCP, especially for those with limited mobility capabilities who rely on caregivers to commute to in-person clinics.

Musculoskeletal pain management guidelines recommend education during interventions [14-16], and digital interventions may play a crucial role in dissemination, given their tailored nature, and wide and convenient accessibility. High engagement in educational content was observed, particularly in older adults.

#### Establishment of a Therapeutic Relationship in Remote Care

Establishing a collaborative relationship between the patient and DPT is key to building rapport, ensuring patient adherence, and driving positive clinical outcomes [51,52]. The DCP ensured collaborative goal setting, development of achievable tasks during onboarding, and ongoing bidirectional communication [51,52]. These factors have been previously shown to be key elements in establishing a strong therapeutic alliance [51-53]. The higher number of messages sent by older adults to the DPT, and the higher satisfaction with the program highlight the importance of the DCP design to change the perception of lack of personal connection in digital care. These results are in line with studies reporting that technologically advanced solutions can achieve the same level of trust as traditional methods [54].

#### Clinical Outcomes

Significant and similar improvements in pain (including comparable response rates) were observed across age groups. Older adults have lower pain thresholds and lower tolerance than their younger counterparts [55], and have been shown to have lower recovery rates on some outcome measures than their younger peers [56]. The higher number of completed sessions by older adults may have contributed to this finding, as higher adherence is associated with better outcomes [57,58]. Despite a larger proportion of older adults reporting analgesic consumption at baseline, they were able to significantly reduce analgesic intake to the same extent as other age groups. This is particularly important in an era where medications are overprescribed and older adults are prone to side effects and drug-drug interactions [59-61].

Musculoskeletal pain is a major driver of productivity impairment [62,63]. At baseline, 79.4% of older adults were in the workforce, but about half reported productivity issues mainly driven by absenteeism [64,65]. Older patients reported similarly significant productivity and non–work-related activity improvements as younger patients at the program end. This suggests that despite the obstacles to returning to work for this age group [66], the DCP was effective in reducing absenteeism. Non–work-related activity improvement is particularly important for older adults as it contributes to the maintenance of autonomy.

Collectively, these findings supported wider dissemination of DCPs in the older adult population. Although not all patients may be eligible for a digital program (eg, due to cognitive decline) [67], a significant proportion of this population could benefit from timely and continuous care to manage their chronic musculoskeletal conditions. Future research should aim to identify and better characterize those who can benefit the most from digital programs, and design and study ways to improve implementation. Mobilizing older adults toward the use of digital technology may empower patients to play an active role in care management, thereby decreasing condition-related mental distress and improving their overall quality of life.

### Strengths and Limitations

The major strength of this study is the novelty of analyzing specific engagement metrics to deep dive into the older adults’ interface with a DCP, which were not explored before. An additional strength is the wide range of clinical outcomes based on validated scales, which can enhance generalizability. This study provides the groundwork to further develop and refine telerehabilitation programs that ensure equitable and continuous care regardless of age.

The major limitation is the lack of a control group, for which the most obvious comparator would be a “waiting list.” This may not be ethical considering the high accessibility this technology affords in a real-world context. Another alternative would be a control group that receives “usual care,” which could provide valuable insight into the acceptance of digital interventions versus conventional care. Since the program enrolled beneficiaries of employers’ health benefits, the current cohort may not be representative of the older adult population in the United States, for whom Medicare is the major insurance payer. Despite education levels being considered a proxy of digital literacy, other objective metrics might provide a better understanding of the impact of digital literacy on telerehabilitation. Finally, the lack of long-term follow-up precludes the evaluation of long-term benefits.

### Conclusions

This study reports high adherence, engagement, and satisfaction with a digital musculoskeletal care program in an older adult population, which were greater than in younger counterparts. Older adults achieved statistically significant and clinically meaningful improvements in all studied outcomes (in pain, mental health, analgesics consumption, and productivity), suggesting that DCPs can successfully overcome some of the barriers surrounding participation in this population. This study showcases the importance of acknowledging generational needs when designing digital interventions in order to ensure equitable and continuous care regardless of age.

## Appendix

Multimedia Appendix 1 Information regarding (1) baseline clinical characteristics for unfiltered cases, (2) model estimates and fitness from latent growth curve analysis (LGCA) following intent-to-treat analysis, (3) statistical differences in exercise performance between groups, (4) model estimates and fitness from LGCA stratified by education levels following intent-to-treat analysis, (5) model estimates from the conditional model, (6) model fitness from conditional LGCA, (7) statistical differences between groups in mean changes for clinical outcomes, (8) probability of analgesics consumptions, and (9) medication reduction trajectories per age category.

Multimedia Appendix 2 Overview of the exercise prescription framework, including exercise characteristics, dosage parameters, and progression principles.

## Abbreviations

CBT: cognitive behavioral therapy
DCP: digital care program
GAD-7: Generalized Anxiety Disorder 7-item scale
mLGCA: multiple-group latent growth curve analysis
OR: odds ratio
PHQ-9: Patient Health Questionnaire 9-item scale
WPAI: Work Productivity and Activity Impairment Questionnaire

## Data Availability

The data sets generated or analyzed during this study are available from the corresponding author upon reasonable request.
