# Addendum: Managing Musculoskeletal Pain in Older Adults Through a Digital Care Solution: Secondary Analysis of a Prospective Clinical Study

**DOI:** 10.2196/97495

**Published:** 2026-08-27

**Authors:** Anabela C Areias, Dora Janela, Maria Molinos, Robert G Moulder, Virgílio Bento, Vijay Yanamadala, Steven P Cohen, Fernando Dias Correia, Fabíola Costa

**Affiliations:** 1 Sword Health, Inc New York, NY United States; 2 Institute for Cognitive Science, University of Colorado Boulder Boulder, CO United States; 3 Department of Surgery, Frank H Netter School of Medicine, Quinnipiac University Hamden, CT United States; 4 Department of Neurosurgery, Hartford Healthcare Medical Group Westport, CT United States; 5 Departments of Anesthesiology & Critical Care Medicine, Physical Medicine and Rehabilitation, Neurology, and Psychiatry and Behavioral Sciences, Johns Hopkins School of Medicine Baltimore, MD United States; 6 Departments of Anesthesiology and Physical Medicine and Rehabilitation, Uniformed Services University of the Health Sciences Bethesda, CT United States; 7 Neurology Department, Centro Hospitalar e Universitário do Porto Porto Portugal

The authors of the publication “Managing Musculoskeletal Pain in Older Adults Through a Digital Care Solution: Secondary Analysis of a Prospective Clinical Study” [1] have introduced further details regarding the exercise component of the intervention to improve transparency and interpretability. To provide a generalized framework underlying the exercise prescription within the digital care program, Multimedia Appendix 2 was added (included here as Multimedia Appendix 1), including representative exercise categories, progression principles, and dosage parameter ranges. Additionally, a detailed description of a condition-specific implementation of digital care programs was reported in a previously published study [2], cited in the Introduction section of the original article. These clarifications do not impact the results or conclusions of the original study.

## Appendix

Multimedia Appendix 1 Overview of the exercise prescription framework, including exercise characteristics, dosage parameters, and progression principles.
