# Supplementary material for: Managing Musculoskeletal Pain in Older Adults Through a Digital Care Solution: Secondary Analysis of a Prospective Clinical Study
Source: JMIR Rehabil Assist Technol. 2023 Aug 15;10:e49673. doi: 10.2196/49673 (PMC10466151; doi:10.2196/49673)
Supplement: Multimedia Appendix 1 [file rehab_v10i1e49673_app1.docx]

# Supplementary Materials

# Managing musculoskeletal pain in older adults through a digital care solution: a secondary analysis of a prospective clinical study

Anabela C. Areias, PhD^1^; Dora Janela^1^, PT; Maria Molinos^1^, PhD; Robert Moulder^1,2^, PhD; Vírgilio Bento^1^, PhD; Vijay Yanamadala^1,3,4^, MD, MBA; Steven P. Cohen^5,6^, MD, PhD; Fernando Dias Correia^1,7^, MD, PhD; Fabíola Costa^1^, PhD;

^1^SWORD Health Inc, Draper, UT, United States

^2^Institute for Cognitive Science, University of Colorado, Boulder, CO, United States

^3^Department of Surgery, Frank H Netter School of Medicine, Quinnipiac University, Hamden, CT, United States

^4^Department of Neurosurgery, Hartford Healthcare Medical Group, Westport, CT, United States

^5^Department of Anesthesiology and Critical Care Medicine, Johns Hopkins School of Medicine, Baltimore, MD, United States

^6^Department of Physical Medicine and Rehabilitation, Uniformed Services University of the Health Sciences, Bethesda, MD, United States

^7^Neurology Department, Centro Hospitalar e Universitário do Porto, Porto, Portugal

## **Table S1.** Clinical characteristics of overall population at baseline stratified by age for unfiltered cases.

| Outcomes | Age group (years) | | | | | | |
| --- | --- | --- | --- | --- | --- | --- | --- |
|  | Young Adults (≤44) | | Middle-aged Adults (45-64) | | Older adults ( ≥65) | |  |
|  | N | Mean (SD) | N | Mean (SD) | N | Mean (SD) | *P* value |
| GAD-7 | 4628 | 4.09 (4.9) | 6726 | 2.87 (4.2) | 727 | 2.06 (3.4) | <.001 |
| PHQ-9 | 4628 | 3.17 (4.8) | 6726 | 2.35 (4.1) | 727 | 2.02 (3.4) | <.001 |
| WPAI - Overall | 4000 | 19.1 (22.9) | 5778 | 17.2 (22.5) | 541 | 15.4 (22.5) | <.001 |
| WPAI - Work | 3967 | 17.3 (20.4) | 5717 | 15.4 (19.8) | 528 | 12.6 (17.8) | <.001 |
| WPAI - Time | 4000 | 2.8 (12.4) | 5778 | 2.7 (12.6) | 541 | 3.4 (16.2) | .41 |
| WPAI - Activity | 4628 | 26.7 (23.9) | 6726 | 27.1 (24.9) | 727 | 25.9 (24.6) | .36 |

## **Table S2.** Model estimates and fitness from unconditional latent growth curve analysis stratified by age categories for exercise performance: Intention-to-treat.

| Intercept  Mean (95%CI) | *P* | Slope  Mean (95%CI) | *P* | Curve  Mean (95%CI) | *P* |
| --- | --- | --- | --- | --- | --- |
| Group ≤44 | | | | | |
| 94.53  (94.3; 94.8) | <.001 | 0.17  (0.13; 0.21) | <.001 | 0.004  (0.006; 0.003) | <.001 |
| Group 45-64 | | | | | |
| 93.50  (93.3; 93.7) | <.001 | 0.18  (0.15; 0.20) | <.001 | 0.004  (0.005; 0.003) | <.001 |
| Group ≥65 | | | | | |
| 91.5  (90.8; 92.2) | <.001 | 0.28  (0.20; 0.35) | <.001 | 0.005  (0.007; 0.003) | <.001 |
| Note: Model fit results (Chi-square: 5573.9, P<.001, CFI: 0.95; TLI: 0.95; RMSEA: 0.02; SRMR: 0.41)  If a significant chi-square is found for a model, then CFI values > .9, or RMSEA values < .08, or SRMR values <.05 signify models with acceptable fit [40,41]. | | | | | |

## **Table S3.** Statistical differences in exercise performance between groups.

| Intercept | ≤44 | 45-64 | ≥65 | Slope | ≤44 | 45-64 | ≥65 |
| --- | --- | --- | --- | --- | --- | --- | --- |
| ≤44 |  | <.001 | <.001 | ≤44 |  | .81 | .009 |
| 45-64 |  |  | <.001 | 45-64 |  |  | .009 |
| ≥65 |  |  |  | ≥65 |  |  |  |
| Curvature | ≤44 | 45-64 | ≥65 |  | | | |
| ≤44 |  | .39 | .65 |  |  |  |  |
| 45-64 |  |  | .28 |  |  |  |  |
| ≥65 |  |  |  |  |  |  |  |

## **Table S4.** Model estimates and fitness from conditional latent growth curve analysis stratified by education levels for older adults for exercise performance: Intention-to-treat.

| Education category | Intercept  Mean (95%CI) | *P* | Slope  Mean (95%CI) | *P* | Curve  Mean (95%CI) | *P* |
| --- | --- | --- | --- | --- | --- | --- |
| Lower education levels | 91.71  (90.58; 92.85) | <.001 | 0.25  (0.14; 0.36) | <.001 | -0.005  (-0.008; -0.002) | <.001 |
| Higher education levels | 91.80  (90.89; 92.71) | <.001 | 0.28  (0.19; 0.38) | <.001 | -0.005  (-0.008; -0.003) | <.001 |
| Note: Model fit results (Chi-square: 1644.1, P=0.00, CFI: 0.75; TLI: 0.78; RMSEA: 0.05; SRMR: 0.08) [40,41]. | | | | | | |

## **Table S5.** Model estimates from conditional latent growth curve analysis stratified by age categories for the clinical outcomes: Intention-to-treat.

| Outcome | Intercept  Mean (95%CI) | *P* | Slope  Mean (95%CI) | *P* | Curve  Mean (95%CI) | *P* |
| --- | --- | --- | --- | --- | --- | --- |
| Group ≤44 | | | | | | |
| Pain Level | 4.26  (4.12; 4.41) | <.001 | -0.38  (-0.44;-0.32) | <.001 | 0.01  (0.01; 0.02) | <.001 |
| GAD-7 ≥5 | 9.03  (8.46; 9.60) | <.001 | -1.07  (-1.30; -0.84) | <.001 | 0.05  (0.03; 0.07) | <.001 |
| GAD-7 | 3.06  (2.70; 3.42) | <.001 | -0.32  (-0.41;-0.22) | <.001 | 0.01  (0.01; 0.02) | <.001 |
| PHQ-9 ≥5 | 9.22  (8.56; 9.88) | <.001 | -1.09  (-1.40; -0.78) | <.001 | 0.06  (0.03; 0.08) | <.001 |
| PHQ-9 | 2.61  (2.27; 2.95) | <.001 | -0.27  (-0.36;-0.17) | <.001 | 0.01  (0.01; 0.02) | <.001 |
| WPAI - Overall >0 | 29.72  (27.39; 32.04) | <.001 | -2.76  (-3.72; -1.79) | <.001 | 0.12  (0.04; 0.20) | <.001 |
| WPAI - Overall | 16.75  (14.96; 18.54) | <.001 | -1.30  (-1.91; -0.69) | <.001 | 0.05  (0.00; 0.10) | .05 |
| WPAI - Activity >0 | 31.94  (30.14; 33.75) | <.001 | -3.72  (-4.46; -2.98) | <.001 | 0.17  (0.11; 0.23) | <.001 |
| WPAI - Activity | 23.91  (22.15; 25.66) | <.001 | -2.37  (-2.99; -1.74) | <.001 | 0.09  (0.05; 0.14) | <.001 |
| WPAI - Work >0 | 26.81  (24.79; 28.83) | <.001 | -3.14  (-4.05; -2.24) | <.001 | 0.16  (0.08; 0.24) | <.001 |
| WPAI - Work | 14.91  (13.32; 16.49) | <.001 | -1.41  (-1.98; -0.84) | <.001 | 0.07  (0.02; 0.11) | <.001 |
| WPAI - Time >0 | 23.63  (21.17; 26.08) | <.001 | -4.00  (-4.93; -3.08) | <.001 | 0.24  (0.16; 0.31) | <.001 |
| WPAI - Time | 2.88  (1.87; 3.89) | <.001 | -0.03  (-0.40; 0.34) | .88 | -0.01  (-0.04; 0.02) | .56 |
| Group 45-64 | | | | | | |
| Pain Level | 4.70  (4.59; 4.82) | <.001 | -0.43  (-0.48; -0.39) | <.001 | 0.02  (0.01; 0.02) | <.001 |
| GAD-7 ≥5 | 8.25  (7.77; 8.74) | <.001 | -0.99  (-1.20; -0.79) | <.001 | 0.05  (0.04; 0.07) | <.001 |
| GAD-7 | 2.06  (1.82; 2.29) | <.001 | -0.20  (-0.26; -0.13) | <.001 | 0.01  (0.00; 0.01) | <.001 |
| PHQ-9 ≥5 | 8.07  (7.51; 8.63) | <.001 | -0.89  (-1.16; -0.63) | <.001 | 0.04  (0.02; 0.06) | <.001 |
| PHQ-9 | 1.76  (1.55-1.98) | <.001 | -0.15  (-0.21; -0.09) | <.001 | 0.01  (0.00; 0.01) | .01 |
| WPAI - Overall >0 | 29.02  (27.10; 30.93) | <.001 | -3.69  (-4.50; -2.88) | <.001 | 0.18  (0.11; 0.25) | <.001 |
| WPAI - Overall | 14.18  (12.82; 15.54) | <.001 | -1.37  (-1.85; -0.90) | <.001 | 0.06  (0.02; 0.10) | <.001 |
| WPAI - Activity >0 | 31.61  (30.10; 33.12) | <.001 | -4.00  (-4.59; -3.42) | <.001 | 0.20  (0.15; 0.25) | <.001 |
| WPAI - Activity | 22.24  (20.82; 23.67) | <.001 | -2.40  (-2.88; -1.91) | <.001 | 0.11  (0.07; 0.15) | <.001 |
| WPAI - Work >0 | 26.42  (24.72; 28.11) | <.001 | -3.77  (-4.51; -3.03) | <.001 | 0.19  (0.13; 0.25) | <.001 |
| WPAI - Work | 12.40  (11.18; 13.62) | <.001 | -1.30  (-1.72; -0.87) | <.001 | 0.06  (0.02; 0.09) | <.001 |
| WPAI - Time >0 | 25.96  (23.51; 28.41) | <.001 | -3.50  (-4.23; -2.77) | <.001 | 0.17  (0.11; 0.23) | <.001 |
| WPAI - Time | 2.31  (1.58; 3.04) | <.001 | -0.14  (-0.40; 0.13) | .31 | 0.01  (-0.02; 0.03) | .59 |
| Group ≥65 | | | | | | |
| Pain Level | 4.63  (4.22; 5.04) | <.001 | -0.39  (-0.53; -0.25) | <.001 | 0.02  (0.01; 0.03) | <.001 |
| GAD-7 ≥5 | 8.00  (6.30; 9.70) | <.001 | -0.56  (-1.24; 0.11) | .10 | 0.03  (-0.03; 0.08) | .38 |
| GAD-7 | 2.18  (1.47; 2.90) | <.001 | -0.08  (-0.30; 0.15) | .52 | 0.00  (-0.02; 0.02) | 1.00 |
| PHQ-9 ≥5 | 6.89  (5.63; 8.15) | <.001 | -0.59  (-1.22; 0.03) | .06 | 0.03  (-0.02; 0.09) | .20 |
| PHQ-9 | 2.05  (1.41; 2.69) | <.001 | -0.13  (-0.35; 0.09) | .26 | 0.01  (-0.01; 0.02) | .37 |
| WPAI - Overall >0 | 25.10  (19.50; 30.70) | <.001 | -4.74  (-7.05; -2.44) | <.001 | 0.35  (0.14; 0.55) | <.001 |
| WPAI - Overall | 12.61  (8.38; 16.84) | <.001 | -2.32  (-3.72; -0.92) | <.001 | 0.18 (  0.06; 0.30) | <.001 |
| WPAI - Activity >0 | 25.81  (20.90; 30.73) | <.001 | -2.69  (-4.38; -1.01) | <.001 | 0.13  (0.00; 0.26) | .05 |
| WPAI - Activity | 16.48  (12.09; 20.88) | <.001 | -1.34  (-2.69; 0.01) | .05 | 0.07  (-0.04; 0.17) | .22 |
| WPAI - Work >0 | 22.35  (17.95; 26.74) | <.001 | -3.01  (-4.38; -1.01) | <.001 | 0.18  (0.03; 0.34) | .02 |
| WPAI - Work | 11.30  (7.90; 14.69) | <.001 | -1.45  (-2.66; -0.24) | .02 | 0.09  (0.00; 0.18) | .05 |
| WPAI - Time >0 | 37.33  (26.39; 48.26) | <.001 | -8.14  (-11.46; -4.82) | <.001 | 0.51  (0.31; 0.72) | <.001 |
| WPAI - Time | 1.83  (-1.12; 4.78) | .22 | -0.85  (-1.75; 0.05) | .06 | 0.08  (0.00; 0.17) | .06 |

## **Table S6.** Model fitness from conditional latent growth curve analysis for the clinical outcomes.

|  | Fit | | | | |
| --- | --- | --- | --- | --- | --- |
| Outcome | Chi-sq (df) | *P* | RMSEA | CFI | SRMR |
| Pain Level | 291.52 (21) | <.001 | 0.057 | 0.95 | 0.020 |
| GAD-7 ≥5 | 21.00 (21) | <.001 | 0.050 | 0.97 | 0.020 |
| GAD-7 | 61.83 (21) | <.001 | 0.022 | 1.00 | 0.007 |
| PHQ-9 ≥5 | 21.00 (21) | <.001 | 0.050 | 0.97 | 0.020 |
| PHQ-9 | 53.02 (21) | <.001 | 0.020 | 1.00 | 0.007 |
| WPAI - Overall >0 | 21.00 (21) | <.001 | 0.030 | 0.98 | 0.010 |
| WPAI - Overall | 39.39 (21) | .01 | 0.015 | 1.00 | 0.008 |
| WPAI - Activity >0 | 21.00 (21) | <.001 | 0.030 | 0.98 | 0.010 |
| WPAI - Activity | 44.35 (21) | <.001 | 0.017 | 1.00 | 0.008 |
| WPAI - Work >0 | 21.00 (21) | <.001 | 0.030 | 0.98 | 0.020 |
| WPAI - Work | 35.21 (21) | .03 | 0.013 | 1.00 | 0.009 |
| WPAI - Time Missed >0 | 3.00 (21) | <.001 | 0.084 | 0.97 | 0.028 |
| WPAI - Time Missed | 19.66 (21) | .54 | 0.000 | 1.00 | 0.006 |
| Abbreviations: GAD-7, Generalized Anxiety Disorder 7-item scale; PHQ-9, Patient Health 9-item questionnaire; WPAI, Work Productivity and Activity Impairment Questionnaire. If a significant chi-square is found for a model, then CFI values > .9, or RMSEA values < .08, or SRMR values <.05 signify models with acceptable fit [40,41]. | | | | | |

###

## **Table S7.** Statistically differences between groups in mean changes for all clinical outcomes.

| Outcome | Young Adults | Middle-aged Adults | Older Adults | Outcome | Young Adults | | Middle-aged Adults | | Older Adults |
| --- | --- | --- | --- | --- | --- | --- | --- | --- | --- |
| Pain |  | | | WPAI overall>0 |  | | | | |
| Young Adults |  | -0.25  (-0.60;0.10) *P*=.16 | 0.26  (-0.39;0.91) *P*=.43 | Young Adults |  | | -2.28  (-7.41;2.84)  *P*=.38 | | 8.89  (-2.43;20.20)  *P*=.12 |
| Middle-aged Adults |  |  | 0.51  (-0.10;1.12) *P*=.10 | Middle-aged Adults |  | |  | | 11.17  (0.22;22.13)  *P*=.05 |
| PHQ-9≥5 |  | | | WPAI  Work>0 |  | | | | |
| Young Adults |  | 0.04  (-1.72;1.80) *P*=.97 | 2.87  (-0.28;6.03)  *P*=.07 | Young Adults |  | | -3.13  (-7.72;1.47)  *P*=.18 | | 4.93  (-2.67;12.53)  *P*=.20 |
| Middle-aged Adults |  |  | 2.84  (-0.14;5.81)  *P*=.06 | Middle-aged Adults |  | |  | | 8.06  (1.03; 15.08)  *P*=.02 |
| GAD-9≥5 |  | | | WPAI  Time>0 |  | | | | |
| Young Adults |  | 0.95  (-0.48;2.38)  *P*=.19 | 2.11  (-0.26;4.48)  *P*=.08 | Young Adults |  | | -3.71  (-8.99;1.57)  *P*=.17 | | -9.75  (-20.77;1.26)  *P*=.08 |
| Middle-aged Adults |  |  | 1.17  (-1.16;3.49)  *P*=.33 | Middle-aged Adults |  | |  | | -6.04  (-16.81;4.74)  *P*=.27 |
|  | | | | WPAI  Activity>0 | |  | | | |
|  |  |  |  | ≤44 | |  | | 1.35  (-2.41;5.12)  P=.48 | 7.08  (-0.17;14.33)  *P*=.06 |
|  |  |  |  | Middle-aged Adults | |  | |  | 5.72  (-1.23;12.68)  *P*=.11 |

## **Table S8.** Probability of consuming analgesics at baseline and program end, and corresponding significant levels for mean changes between groups.

| Age group | Baseline probability  Mean (95%CI) | *P* value | End-program probability  Mean (95%CI) | *P* value | *P* values for mean changes between groups | | |
| --- | --- | --- | --- | --- | --- | --- | --- |
| Group ≤44 | 0.159  (0.157; 0.160) | <.001 | 0.119  (0.117; 0.120) | <.001 | .96 | .36 |  |
| Group 45-64 | 0.272  (0.270; 0.275) | <.001 | 0.216  (0.214; 0.218) | <.001 |  |  | .30 |
| Group ≥65 | 0.352  (0.349; 0.354) | <.001 | 0.260  (0.258; 0.263) | <.001 |  |  |  |

Note: Model fit results (Chi-square: 25.4, P<.001, CFI: 0.99; TLI: 0.99; RMSEA: 0.054; SRMR: 0.020)


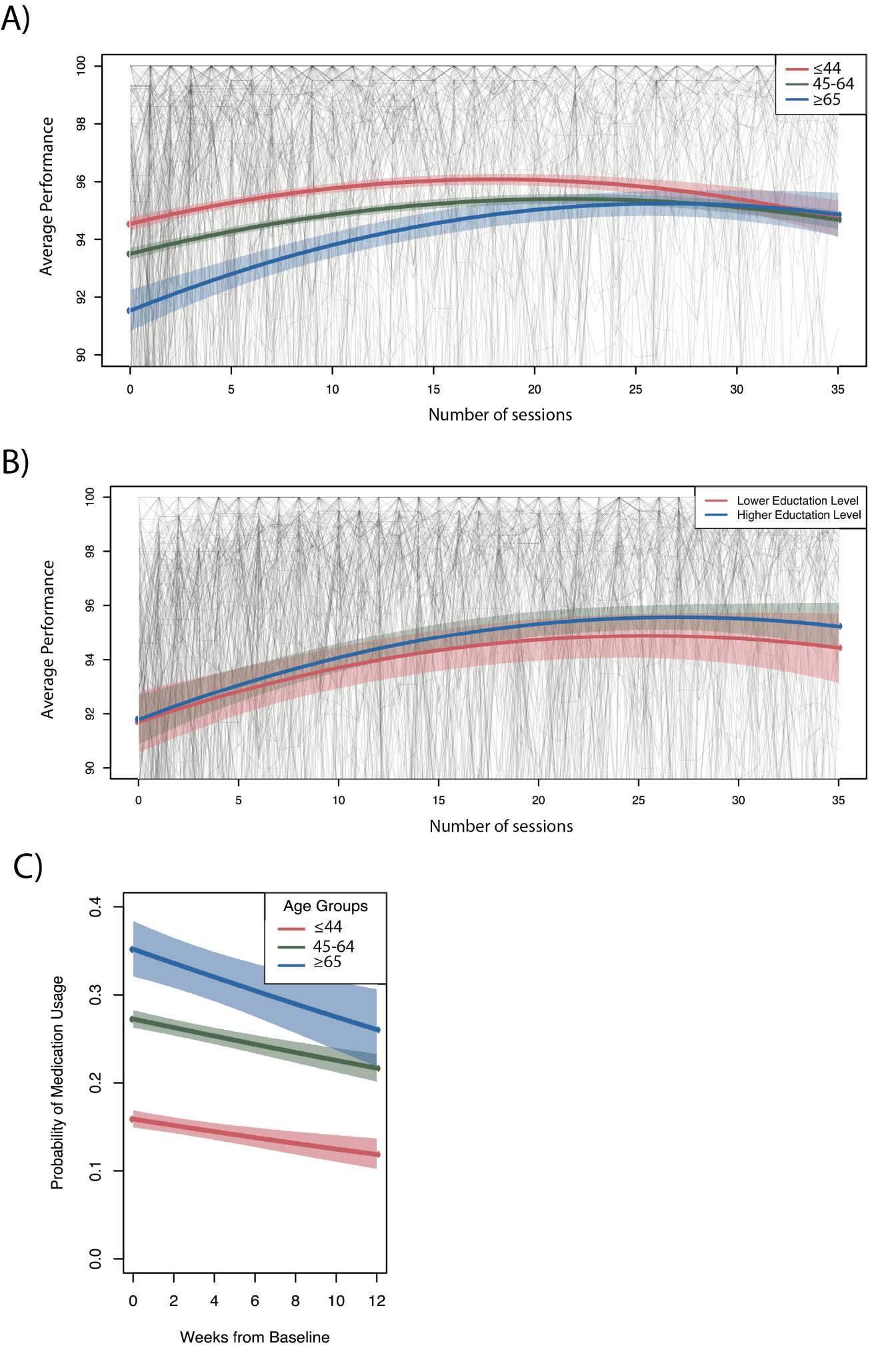


## **Figure S1.** Medication reduction trajectories per age category. Shadowing indicates each trajectory confidence interval, while individual trajectories are depicted with lighter gray lines.
