# Supplementary material for: Managing Musculoskeletal Pain in Older Adults Through a Digital Care Solution: Secondary Analysis of a Prospective Clinical Study
Source: JMIR Rehabil Assist Technol. 2023 Aug 15;10:e49673. doi: 10.2196/49673 (PMC10466151; doi:10.2196/49673)
Supplement: Supplementary file 2 [file rehab_v10i1e49673_app2.docx]

**Table 1.** Overview of the exercise prescription framework, including exercise  characteristics, dosage parameters, and progression principles.

| **Main Goals** | **Description** |
| --- | --- |
| **Initial Phase** ·    Reduce symptom burden (e.g., pain, discomfort) ·    Encourage gradual reintroduction of previously avoided movements ·    Improve joint mobility and movement capacity ·    Initiate low-load strengthening and motor control exercises | Programs typically included 1-2 sets per exercise, which included a combination of: ·  Articular mobility exercises (e.g., sitting trunk flexion and extension; cat-camel exercise) ·  Strengthening exercises (e.g., sit-to-stand, wall push-ups) ·  Active and passive stretching exercises (e.g., seated hamstring stretch, levator scapula stretch) ·  Condition-specific techniques when appropriate (e.g., tendon gliding exercises) |
| **Intermediate Phase** ·    Further improve mobility and flexibility ·    Progress muscular strength and endurance ·    Increase tolerance to movement and activity-related symptoms ·    Gradual reintroduction of functional and daily-life activities | Exercise programs were adapted based on patient response, typically by increasing the range of motion, exercise dosage (sets, repetitions, session duration) or load (e.g., add external load or modifying bodyweight load). Aim for at least 2-3 sets of: ·  Articular mobility exercises (e.g., standing trunk movements, combined trunk flexion and rotation) ·  Strengthening exercises (e.g., isometric wall squat, prone press-ups;) ·  Active and passive stretching exercises (e.g., standing hamstring stretch) |
| **Late Phase** ·    Restore functional capacity ·    Return to usual daily and occupational activities ·    Promote long-term self-management and independence | Add or increase exercise dosage (sets, repetitions, session duration) or load, increase task complexity (e.g., stability demands) and add single leg stance exercises: ·  Articular mobility exercises (e.g., single leg knee to chest) ·  Strengthening exercises (e.g., plank with trunk or hand movement; step up) ·  Active and passive stretching exercises |

***Note:*** *Exercise prescription and progression was individualized based on baseline clinical assessment, ongoing feedback from the patient, performance metrics captured by the motion tracking technology during sessions, and symptom evolution (pain, fatigue, functional capacity).* *Typical Dosage (illustrative):*

*·* 	*Recommended frequency: 3 sessions per week*

*·* 	*Treatment duration: up to 12 weeks*

*·* 	*Repetition ranges commonly varied (e.g., 8–15 reps depending on goal)*

*·* 	*Programs adjusted dynamically based on tolerance and progression.*
